# Aerogels—Preparation and Properties

**DOI:** 10.3390/gels11120975

**Published:** 2025-12-03

**Authors:** Stoyan Gutzov, Xiaodong Wang

**Affiliations:** 1 Department of Physical Chemistry, Faculty of Chemistry and Pharmacy, Sofia University “St. Kliment Ohridski”, 1164 Sofia, Bulgaria; sgutzov@chem.uni-sofia.bg; 2 School of Physics Science and Engineering, Tongji University, Shanghai 200092, China

## 1. Introduction

Aerogels are ultra-light, nanoporous solid materials characterized by extremely high porosity, with up to 99.8% of their volume consisting of void space. They exhibit exceptionally low thermal conductivity, making them among the most efficient solid insulators known. Their high surface area and translucent appearance contribute to unique catalytic, adsorption, and light-scattering properties.

The first aerogels were synthesized nearly a century ago by S. S. Kistler [1], who employed supercritical drying with carbon dioxide. Since then, a wide variety of aerogels—ranging from bulk monoliths to granules, nanopowders, and thin films—have been developed using diverse synthetic strategies [2,3,4,5]. Almost three decades ago, a physicochemical approach for producing hydrophobic aerogel powders and granules via subcritical drying was reported [6]. This method preserves the pore structure of the gelled metal oxide network by replacing the original solvent with a low-surface-tension liquid such as ethanol. Subsequent surface modification with hydrophobic agents, such as trimethylchlorosilane, enables controlled solvent removal while protecting the delicate pore network. The final drying step is performed under subcritical conditions—typically around 0.1 atm, or even at ambient pressure and near room temperature—making the process considerably milder than conventional supercritical drying. Aerogels produced through this method have been widely utilized as catalysts, optical composites, and thermal insulation fillers [7]. Optical composites with tailored composition, structure, porosity, and luminescent properties suitable for sensing applications have also been obtained using subcritically dried powders [8].

Aerogels encompass metal oxides, semiconductors, carbon, graphene, organic polymers, and hybrid or composite systems, exhibiting a broad spectrum of thermal [9], electrical [10], optical [11], and mechanical properties [12]. Current research increasingly focuses on enhancing the mechanical robustness of aerogels while maintaining their nanoporous microstructure. An emerging direction in aerogel fabrication is three-dimensional (3D) printing [13], which enables precise control over microstructure and holds considerable potential for applications in electronics and biomedical engineering. These rapid developments underline the growing diversity of aerogel research, from sustainable precursors to functionally tailored architectures [14]. They also highlight the need for systematic evaluations of new preparation strategies and structure–property relationships. The studies collected in this Special Issue reflect these evolving trends, as summarized in the following section.

## 2. Overview of the Publications in This Special Issue

This Special Issue compiles eleven high-quality research articles that collectively advance the state of the art in aerogel science. The contributed works span critical areas, from the synthesis of novel functional aerogels and the development of sustainable precursors to the use of advanced Machine Learning (ML) techniques for property prediction and optimization.

The first study, “Porous Silica Gels Doped with Gold Nanoparticles: Preparation, Microstructure, Optical and Textural Properties” [contribution 1], detailed the synthesis of porous silica gel powders doped with gold nanoparticles (AuNPs) by heating silica gels containing 1-dodecanethiol and tetrachloroauric acid at 450–900 °C. Comprehensive characterization via XRD, TEM/EDS, UV/Vis reflectance spectroscopy, and DTA/TG revealed that the color and microstructure of SiO_2_:AuNPs (≈0.03% Au) depend critically on heating temperature. UV/Vis spectra were explained using Mie’s theory, while DTA/TG tracked thermal stability and sol–gel matrix evolution. Pore structure analysis showed high specific surface areas (S_BET_ = 200–350 m^2^/g) and mean pore diameters of ~10 nm. These nanocomposites hold dual promise: as ceramic glaze pigments and as catalytic candidates owing to their aerogel-like porosity.

The second work, “Development of Porous Silicon (Si) Anode Through Magnesiothermic Reduction of Mesoporous Silica (SiO_2_) Aerogel for All-Solid-State Lithium-Ion Batteries” [contribution 2], tackled the volume expansion challenge of Si anodes in all-solid-state lithium-ion batteries (ASSLBs). Using magnesiothermic reduction (MTR) at 600–900 °C, the authors fabricated porous Si anodes from hydrophobic/hydrophilic SiO_2_ aerogels. Characterization confirmed successful Si production with a retained porous structure. Coin cell tests evaluated electrochemical performance: charge/discharge cycling at 1 C (0.02–2 V vs. Li) revealed impacts of silicon content, wettability, and interfacial compatibility, while long-term cycling (1 C, 0–1.5 V vs. Li) assessed capacity retention. This approach underscores the potential of SiO_2_-derived porous Si aerogels to enhance ASSLB durability.

In “Preliminary Investigation into the Use of Amino-Acid-Derived Ionic Liquids for Extracting Cellulose from Waste Biomass to Prepare Cellulose Aerogel Adsorbents” [contribution 3], the authors employed a green amino acid-derived ionic liquid ([Cys][NO_3_]) to extract cellulose from lignocellulosic waste. The extracted cellulose was processed into cellulose aerogels (CAs) via conventional protocols. CAs exhibited robust adsorption capacities for various ions (Na^+^, Ca^2+^, Mg^2+^, Cd^2+^) and engine oil, demonstrating preserved cellulose fibrillation and effective mass transfer channels. This study provides a sustainable, straightforward route to cellulose aerogels for wastewater treatment and material recovery.

In the fourth publication, “Thermal Reverse-Engineered Synthesis and Catalytic Activity of Nanogold-Containing Silica Aerogels” [contribution 4], the authors synthesized SiO_2_–AuNP aerogels via the sol–gel method and supercritical CO_2_ drying, using polyvinyl pyrrolidone (PVP) to stabilize AuNPs during gelation. Compared to PVP-free samples (which formed large Au aggregates), PVP-containing aerogels retained monodisperse AuNPs. Critically, the breakdown of large gold clusters into individual nanoparticles was visually confirmed during the thermal treatment of blue samples. This thermal conversion method introduces a novel strategy for creating catalytically active nanogold-containing silica aerogels.

The researchers in “Exploration of Key Factors in the Preparation of Highly Hydrophobic Silica Aerogel from Rice Husk Ash Assisted by Machine Learning” [contribution 5] utilized a decision tree regression model to optimize hydrophobic silica aerogel (HSA) preparation from rice husk ash. Notably, nitric acid-catalyzed HSA achieved a superhydrophobic water contact angle of 159.5°. The study found that moderate increases in hydrophobic modifier concentration enhanced hydrophobicity but reduced porosity, and confirmed that HSA retained hydrophobicity until 500 °C, thus expanding HSA applications using simple, scalable traditional methods.

In the sixth study, “Machine Learning Models for Predicting Thermal Properties of Radiative Cooling Aerogels” [contribution 6], the authors developed an optimized XGBoost model (R^2^ = 0.943) to predict radiative cooling aerogel performance. The model integrated material composition, optical properties, and environmental factors. SHAP analysis identified ZnO modifiers (SHAP value: 1.523), ambient temperature (1.299), and solar irradiance (0.979) as the most influential factors. Feature interaction analysis elucidated complex composition–environment interplay, guiding efficient radiative cooling material design to address global climate and energy challenges.

In “Lignin Polyurethane Aerogels: Influence of Solvent on Textural Properties” [contribution 7], the authors used organosolv lignin to synthesize polyurethane aerogels. Solvent choice was demonstrated to be the key factor regulating microstructure: high-affinity solvents yielded dense materials, while low-affinity solvents produced aggregated, macroporous structures. Optimally, mixed solvents (e.g., 75% DMSO/acetone) balanced gelation and phase separation, yielding mechanically stable aerogels with a low envelope density (0.49 g/cm^3^) and ~300 m^2^/g specific surface area. This work demonstrates a versatile approach to tailoring lignin polyurethane aerogels’ textural and mechanical properties via simple solvent adjustments.

The authors of “Machine Learning Techniques to Analyze the Influence of Silica on the Physico-Chemical Properties of Aerogels” [contribution 8] applied principal component analysis (PCA) to resolve the effects of silica content on aerogel properties. PCA revealed that the first principal component (PC1) had a strong positive correlation with silica content (R^2^ = 94%), linking higher silica to lower thermal conductivity, porosity, and BET surface area, but higher density and elastic modulus. Additionally, the analysis identified the critical role of thermal conductivity in the second principal component (PC2), particularly in samples with moderate to high silica content. This study showcases the utility of machine learning in optimizing silica aerogel design by unraveling complex property interrelationships.

In the ninth publication, “Synthesis of Flexible Polyamide Aerogels Cross-Linked with a Tri-Isocyanate [contribution 9]”, researchers synthesized flexible polyamide (PA) aerogels using a tri-isocyanate (Desmodur N3300A) as a cross-linker. Supercritical CO_2_ drying yielded aerogels with 19–27% shrinkage, densities of 0.12–0.22 g/cm^3^, porosities up to 91%, specific surface areas of ~309 m^2^/g, and moduli ranging from 20.6 to 109 MPa. These aerogels exhibit greater flexibility than previously reported TPC-mPDA-BTC PA, N3300A-polyimide, and N3300-reinforced silica aerogels, expanding the utility of soft aerogels.

The authors of “Facile Synthesis of Surface-Modified Hollow-Silica Aerogel Particles via Oil–Water–Oil Double Emulsion Method [contribution 10]” used an oil–water–oil double emulsion to encapsulate a volatile oil phase in silica shells, producing surface-modified hollow SiO_2_ aerogel particles. The study optimized key parameters such as O/W ratio, silica concentration, and surfactant concentration. Characterization confirmed hollow microstructures and precise control over particle characteristics. This simple method enhances application potential in drug delivery, catalysis, and insulation by enabling customizable surface properties.

Finally, the study presented in “Enhancing Water Resistance in Foam Cement through MTES-Based Aerogel Impregnation [contribution 11]” addressed the inherent water absorption issue of foam cement by impregnating MTES-based aerogels. MTES-aerogel-enhanced foam cement exhibited 86% lower water absorption than traditional foam cement, with a softening coefficient exceeding 0.75. This method successfully avoids the strength decline associated with direct aerogel incorporation while significantly improving hydrophobicity and water resistance, thereby extending the service life of foam cement and demonstrating the potential of aerogel in construction materials.

## 3. Conclusions

In conclusion, this Special Issue presents cutting-edge studies encompassing a wide range of applications, as well as the synthesis and characterization of aerogel materials with diverse compositions, including organic, hybrid, and inorganic porous crystalline or amorphous species. The applications highlighted span optical materials, catalysts, and electrochemical and biochemical systems. The advanced synthesis strategies discussed enable precise tuning of material properties, opening new avenues for further exploration of the state of matter of aerogel and its potential across multiple scientific and technological fields.
